# Recent Advances in Rare-Earth-Doped Nanoparticles for NIR-II Imaging and Cancer Theranostics

**DOI:** 10.3389/fchem.2020.00496

**Published:** 2020-06-17

**Authors:** Zhenfeng Yu, Christina Eich, Luis J. Cruz

**Affiliations:** Translational Nanobiomaterials and Imaging Group, Department of Radiology, Leiden University Medical Center, Leiden, Netherlands

**Keywords:** NIR-II, rare-earth-doped nanoparticles, modification, *in vitro* and *in vivo* imaging, cancer theranostics

## Abstract

Fluorescence imaging in the second near infrared window (NIR-II, 1,000–1,700 nm) has been widely used in cancer diagnosis and treatment due to its high spatial resolution and deep tissue penetration depths. In this work, recent advances in rare-earth-doped nanoparticles (RENPs)—a novel kind of NIR-II nanoprobes—are presented. The main focus of this study is on the modification of RENPs and their applications in NIR-II *in vitro* and *in vivo* imaging and cancer theranostics. Finally, the perspectives and challenges of NIR-II RENPs are discussed.

## Introduction

**Figure 1:**
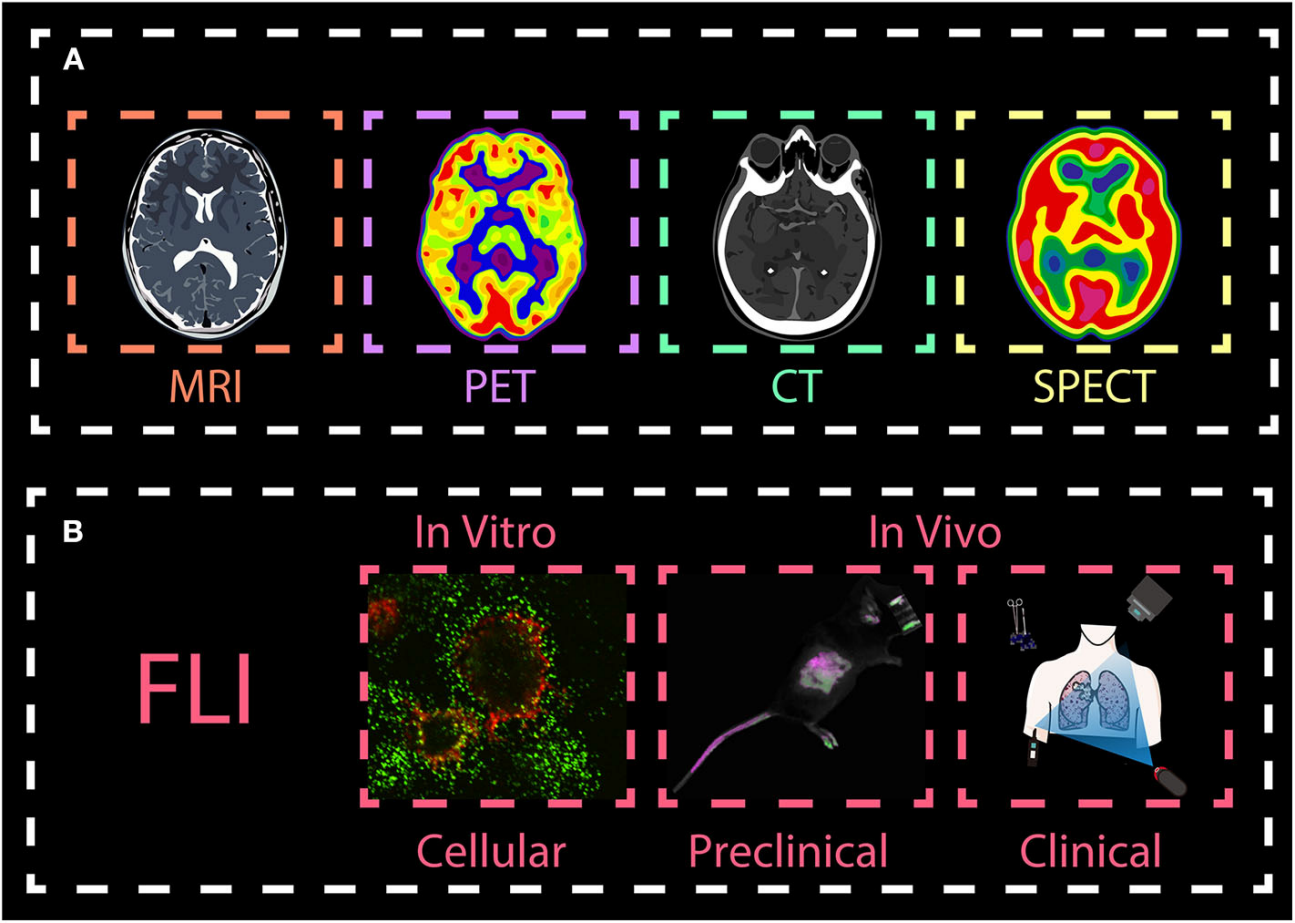
Overview molecular imaging techniques. The main imaging methods are **(A)** magnetic resonance imaging (MRI), X-ray computed tomography (CT), positron emission tomography (PET), single-photon emission tomography (SPECT), and **(B)** optical fluorescent light imaging (FLI). While MRI, PET, CT, and SPECT are widely used in the clinics, FLI techniques are mainly used in biomedical preclinical research *in vitro* and *in vivo*, with the exception of fluorescence image-guided surgery, a medical imaging technique used to detect fluorescently labeled structures during surgery. This review, we will focus on introducing FLI from both *in vitro* and *in vivo* imaging.

Cancer is one of the world's most lethal diseases, and there are no particularly effective treatments to date. Cancer patients must endure chemotherapy and radiotherapy, followed by long-term medications, which are a great burden on their body and mind. For people not to be afflicted by cancer, it is necessary to diagnose the disease in an early stage and personalize treatments based on each patient's individual variability and medical profile (Rubin et al., 2014). Molecular imaging modalities can be useful for the comprehensive evaluation of essential biomolecules and can facilitate the non-invasive visualization of cell function and biochemical processes in biological systems (Kuimova et al., 2009; Weissleder et al., 2016; Yang et al., 2017a). They are well-recognized as powerful techniques that provide more comprehensive anatomical, physiological and functional information in early cancer detection, drug delivery, as well as monitoring treatment effectiveness (Quon and Gambhir, 2005; Weissleder and Pittet, 2008; Willmann et al., 2008). Currently, varieties of molecular imaging techniques are widely used in the medical field, including magnetic resonance imaging (MRI), X-ray computed tomography (CT), positron emission tomography (PET), single-photon emission tomography (SPECT), and optical fluorescent light imaging (FLI) (Figure 1). However, these methods have some disadvantages. For example, CT and MRI often require high doses of contrast agents; PET and SPECT require radioactive tracers that can put both patients and operators in danger (O'Leary et al., 1999; Mariani et al., 2001, 2002; Tsien, 2003). Also, they need to be optimized to obtain more accurate information due to their long scanning time and low sensitivity/spatial resolution (Toussaint et al., 1996; Paulus et al., 2000).

**Figure 2:**
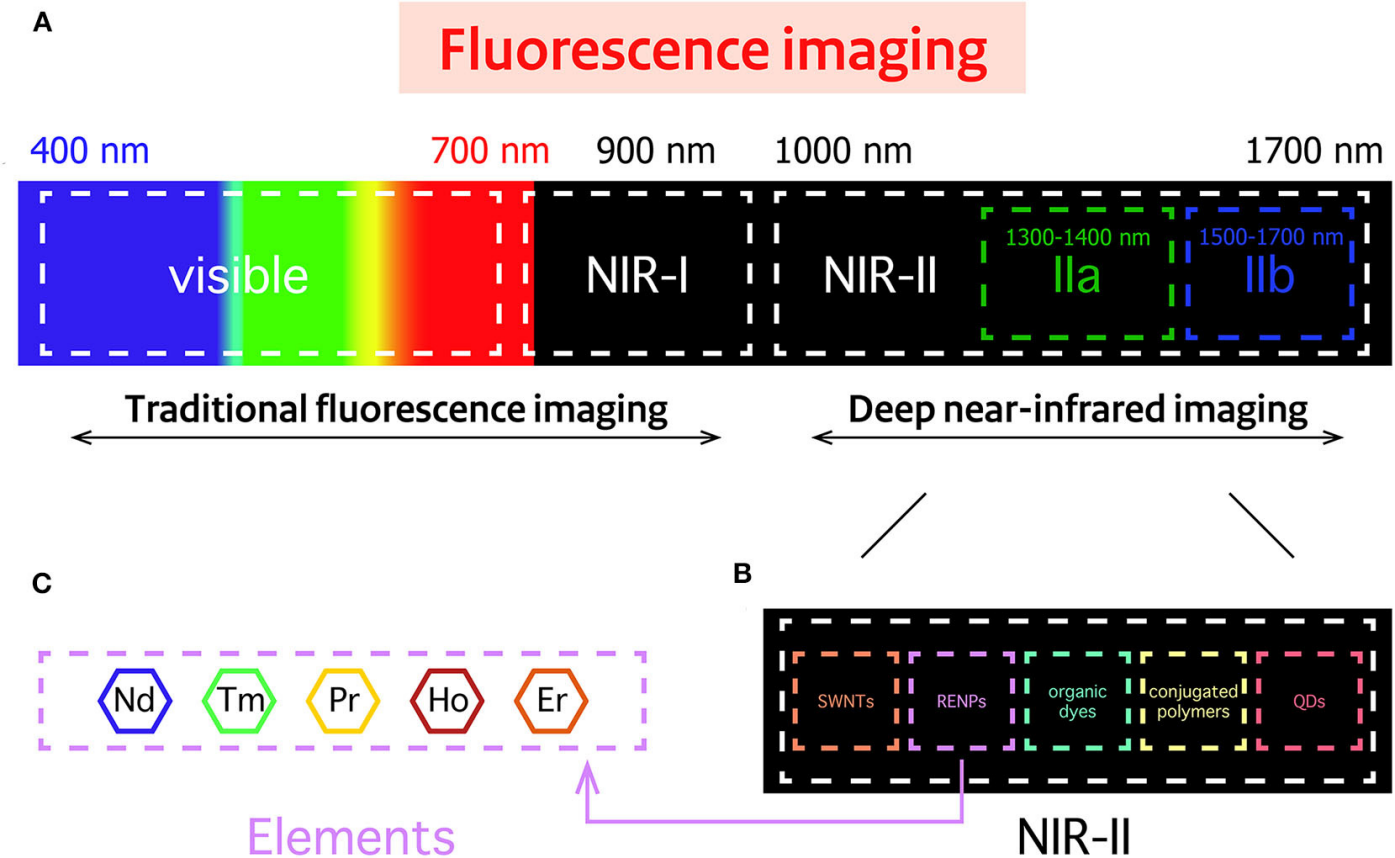
Spectral overview fluorescence imaging techniques. **(A)** Spectral ranges of traditional fluorescence imaging techniques, including the near-infrared (NIR)-I region, and the deep NIR-II region. **(B)** Imaging probes commonly used in the NIR-II region: Single walled nanotubes (SWNTs), rare-earth-doped nanoparticles (RENPs), organic dyes, conjugated polymers, and quantum dots (QDs). **(C)** Neodymium (Nd)-, thulium (Tm)-, praseodymium (Pr)-, holmium (Ho)-, and erbium (Er)-containing RENPs have attracted wide attention.

In recent years, optical imaging has attracted much attention in various fields, predominantly preclinical research because it provides excellent real-time visualization, high sensitivity and spatial resolution, especially in early detection and diagnosis of cancer. Generally, most of the conventional imaging agents operate in the short-wavelength region (e.g., the ultraviolet (UV) and visible regions). In these regions, light signals are easily absorbed and scattered by certain biological tissues (such as muscle, skin and body fluids). This leads to high autofluorescence, low signal-to-background ratio and low tissue penetration (Yang et al., 2017b,c). Besides, high-energy light can lead to photo-toxicity damage in biological tissues. To circumvent these problems, optical imaging in the near-infrared (NIR) region, which is located in the so-called “biological window,” has gained much attention (Figure 2). Imaging agents in the first near-infrared window (NIR-I, 700–900 nm) are gradually being known by researchers, and can provide deep and sensitive bioimaging. However, their limited tissue penetration depth (less to 1 cm) and large photon scattering losses in biological samples still restrict their use further in biomedical diagnosis and therapy. To address these challenges, novel materials that enable fluorescent imaging in the NIR-II window (10,00–1,700 nm) for biomedical applications have been developed. They show better resolution because they have deeper penetration (~1.8 cm) and lower autofluorescence. Therefore, there is need to synthesize the novel NIR-II agents with high efficiency and resolution for biological imaging application (Fan and Zhang, 2019).

So far, many types of fluorescent agents with emission in the NIR-II region have been extensively applied for effective bio-sensing and real-time *in vitro* and *in vivo* imaging of living species (Figure 2). They include single-walled carbon nanotubes (SWNTs) (Gong et al., 2013; Liang et al., 2014), organic dyes (Lei et al., 2019; Wang et al., 2019a), conjugated polymers (Hong et al., 2014), quantum dots (QDs) (Li C. et al., 2014), and rare-earth-doped nanoparticles (RENPs) (Fan et al., 2019; Wang et al., 2019b). However, most of them have some disadvantages, such as the broad emission bandwidths of SWNTs, short accumulation time of organic dyes, high toxicity, low quantum yield and low solubility of QDs. These disadvantages will vastly inhibit their further applications in NIR-II imaging. RENPs are good candidates for NIR-II optical imaging, because they show minimal photo-bleaching, superior luminescent lifetimes, excellent tunable emission wavelengths and low biotoxicity (Rocha et al., 2014; Wang et al., 2014; Dong et al., 2015; Hemmer et al., 2016; Jiang et al., 2016; Kamimura et al., 2017).

Rare earth elements constitute a class of lanthanide ions found in the 6th row of the periodic table (La, Ce, Pr, Nd, Pm, Sm, Eu, Gd, Tb, Dy, Ho, Er, Tm, Yb, Lu), as well as two other elements closely related to the lanthanides—yttrium (Y) and scandium (Sc). Due to the incompletely filled 4f shell and the spin-orbital coupling of 4f free ions, they possess extremely complex optical properties. One of the most interesting features of these ions is their photoluminescence. The luminescence of the trivalent lanthanide ions arises from f-f transitions of the 4f shell and f-d transitions in the 4f-5d shell. The f-f transitions also provide the lanthanide elements with rich energy level structures in the UV, VIS and NIR ranges. As they can be tuned from the UV to the NIR region, most nanomaterials made of rare-earth elements can be classified into two major categories: Upconversion nanoparticles (UCNPs) and downconversion nanoparticles (DCNPs). DCNPs can downconvert a high energy photon into two or more low-energy photons. In contrast, UCNPs can convert long-wavelength light (low energy) to short wavelength (high energy). Unfortunately, due to the unique anti-Stokes optical properties of UCNPs, most of the NIR-II nanoprobes belong to the category of DCNPs. Until now, a large number of studies have shown that by using suitable sensitizers, UCNPs can obtain longer excitation wavelength for NIR-II imaging (Zhang et al., 2020). For example, emissions in the NIR-II region of Ho^3+^ and Nd^3+^ could be obtained from Er^3+^ sensitized UCNPs (Liu et al., 2018).

Based on the excellent characteristics of rare-earth ions, such as their low photobleaching, various absorption and emission wavelengths, and low energy losses, NIR light-mediated RENPs have been widely used in *in vitro* and *in vivo* imaging of biomolecules. Commonly, this kind of downconverting nanoparticle combines rare-earth ions and an inorganic crystalline host lattice (e.g., NaYF_4_, NaLuF_4_, and CaF_2_). The host can also provide an environment for energy transfer from a sensitizer to a rare-earth dopant resulting in NIR-II fluorescence.

This review mainly introduces the recent advances in RENPs fluorescent imaging in the NIR-II region. In particular, we focus on the modification of these nanoparticles by lipids or dyes, and their use in cancer diagnosis and therapy. Then, the challenges and prospects of RENPs are discussed.

## Main Kinds of RENPs

Based on the energy level of the rare earth elements, most RENPs possess upconversion and downconversion properties. Up to date, thanks to the effort of many researchers, five of them are reported and extensively explored as activators emitting in NIR-II regions, having excellent downconversion emission (1,060/1,300 nm for Nd^3+^, 1,470 nm for Tm^3+^, 1,310 nm for Pr^3+^, 1,185 nm for Ho^3+^, and 1,525 nm for Er^3+^) (Liu et al., 2016) (Figure 2).

### Nd-Doped Nanoparticles

According to recent studies, Nd^3+^ has gained attention for bioimaging applications due to its special illumination at 808 nm and deep tissue penetration (Wang et al., 2013). With strong absorption at 730 nm, 808 nm or 860 nm, Nd^3+^ can transfer photons with the generation of electrons from the ^4^I_9/2_ ground state to the ^4^F_7/2_, ^4^F_5/2_, or ^4^F_3/2_; then the electrons move back to the ^4^F_3/2_ state, which can reduce the overheating effect of tissues usually caused by 980 nm light. As a result of the two transitions, the emission corresponds to 1,060 nm (^4^F_3/2_→^4^I_11/2_) and 1,330 nm (^4^F_3/2_→^4^I_13/2_) in the NIR-II region. Thus, it provides a good way to avoid autofluorescence of tissue.

Earlier attempts of using Nd-doped nanomaterials as NIR-II biomedical imaging agents have been described (Villa et al., 2015; Yu et al., 2018). In 2002, Stouwdam et al. first realized that Nd^3+^ doped LaF_3_ nanoparticles can be utilized as a polymer-based optical component under 514 nm laser excitation (Stouwdam and van Veggel, 2002). Then, Wang et al. developed the synthesized method of LaF_3_: Nd^3+^. It was carried out in aqueous solution at low temperature, and showed great NIR-II emission under 802 nm laser excitation (Wang et al., 2006). In 2014, LaF_3_: Nd^3+^ nanoparticles were used to obtain both *in vitro* and *in vivo* images in cancer cells and mice by Rocha et al. The results showed that LaF_3_: Nd^3+^ nanoparticles are a very promising fluorescent nanoprobe for bioimaging in the second NIR window (Rocha et al., 2014). One year later, Villa and his group did an exciting work on high-contrast *in vivo* imaging in the second biological window (Villa et al., 2015). This work showed how to produce autofluorescence free, high contrast *in vivo* fluorescence imaging with 1340 nm emission band of SrF_2_: Nd^3+^ nanoparticles. They found that autofluorescence of animal diet can extend up to about 1,100 nm, which demonstrated that food-related infrared autofluorescence has an impact on the study of reliable biodistribution. In the past 3 years, some new host matrices have been reported, such as LiYF_4_ (Jiang et al., 2016), GdPO_4_ (Yang et al., 2018), CaTiO_3_ (Li et al., 2015), and NaDyF_4_ (Liu et al., 2017). As we know, higher Nd^3+^ doping will result in severe quenching of concentration, so to induce great fluorescence signals, the concentration of Nd^3+^ should be controlled between 1 and 5%. Thanks to intensive research, most of these new Nd^3+^ doped systems are nowadays not only used *in vivo* NIR-II imaging but also in X-ray CT bioimaging or MRI. Owing to the large X-ray absorption coefficient of Gd^3+^, Dy^3+^, dual-mode molecular imaging has become a new trend in bioimaging, such as NIR-II imaging/CT, NIR-II imaging/MRI, NIR-II imaging/PET.

Despite the efforts made so far as seen above, low optical effects are still a major drawback. However, sensitizers and core-shell structures that can be used to increase the signal-to-noise ratio are gradually becoming more known in the field of NIR-II bioimaging, disease detection and treatment. For example, NaGdF_4_: Nd^3+^, Yb^3+^, Tm^3+^ is a novel nanomaterial which uses Gd^3+^ as bridge ions and finally traps energy by the initial activator ions (Nd^3+^) (Zhang et al., 2015). Other previous studies also showed that co-doping with Y^3+^ effectively reduced the aggregation of Nd^3+^ in CaF_2_, resulting in a greater luminescence enhancement of Nd^3+^ (Yu et al., 2018). Chen et al. synthesized high quantum yield core/shell NaGdF_4_: 3%Nd^3+^@NaGdF_4_ nanoparticles with an average size of 15 nm. An *in vitro* and *in vivo* NIR-II bioimaging was obtained by loading HeLa cells with NaGdF_4_: 3%Nd^3+^@NaGdF_4_ nanoparticles and transferring NaGdF_4_: 3%Nd^3+^@NaGdF_4_ nanoparticles in a nude mouse model (Chen et al., 2012). CaF_2_ was also used as the shell material to make NaYF4: Yb, Nd@CaF_2_ core/shell nanoparticles, which resulted in high contrast multiplexed *in vivo* imaging in the NIR-II region (Ortgies et al., 2018). In 2018, inspired by Chen's work, Wang et al. fabricated NaGdF_4_: 5%Nd^3+^@NaGdF_4_ by the successive layer-by-layer (SILAR) method. To obtain DCNPs-L1-FSHβ nanoprobes via an EDC/NHS reaction, image-guided surgery for metastatic ovarian cancer could be improved. Utilizing these novel nanoprobes, metastases with ≤ 1 mm can be completely resected under the guidance of NIR-II imaging (Wang P. et al., 2018). A recent report showed that the ultra-small NaGdF_4_: 5 %Nd@NaGdF_4_ (4.38 ± 0.57 nm) nanoparticles can be applied in the precise inflammation bioimaging by ROS (reactive oxygen species)-responsive cross-linking after modification with GSH (Glu-Cys-Gly) (Wang et al., 2014; Zhao et al., 2019). An interesting work based on supramolecular self-assembly strategy is developed for NIR-II imaging assembly and disassembly through NaGdF_4_: 10%Y, 25%Yb, 0.5%Tm@NaGdF_4_ UCNP@azobenzene and NaGdF_4_: 5%Nd@NaGdF_4_ DCNP@β-cyclodextrin. The new strategy allows flexible assembly and disassembly of nanoparticles by controlling different NIR-lasers, which can reduce the background of biological imaging and long-term cytotoxicity, while providing technical support for further accurate image-guided tumor surgery (Zhao M. et al., 2018). As only a few NIR-II fluorophores can be used directly for bone imaging without linking to targeted ligands, He et al. demonstrated DSPE-mPEG encapsulated with β-phase NaYF_4_: 7%Nd@NaYF_4_ can be used for bone and vascular imaging, even real-time image-guided lymph node mapping and resection (He et al., 2019) (Table 1).

**Table 1:** Typical of Nd-RENPs NIR-II nano-composites.

**NIR-II compositions**	**Excitation wavelength (nm)**	**Emission wavelength (nm)**	**Ligands**	**Applications**
SrF _2_: Nd^3+^	808	900–1,500	−	*In vitro* and *in vivo* NIR-II imaging
CaF_2_: Y^3+^, Nd^3+^	808	1,058	−	*In vivo* NIR-II imaging
LaF _3_: Nd^3+^	808	910, 1,050, 1,330	−	*In vitro* and *in vivo* NIR-II imaging
LiYF_4_: 5%Nd^3+^	808	900, 1,050, 1,330	EDTMP	Bio-imaging and biodetection
GdPO_4_: Nd^3+^	808	1,050, 1,330	DOX	Dual-modal *in vivo* NIR-II/X-ray bioimaging and pH-responsive drug delivery
NaDyF_4_: 10%Nd	808	1,050, 1,330	Gallic acid-Fe(III)	NIR-II imaging, MRI imaging, PTT
NaGdF_4_: Nd^3+^, Yb^3+^, Tm^3+^	800	980, 1,060	−	NIR-II imaging, MRI imaging
NaGdF_4_: Nd^3+^@NaGdF_4_	740/900	1,050, 1,300	−	*In vitro* and *in vivo* NIR-II imaging
NaYF_4_: Yb,Nd@CaF_2_	808	980, 1,350	Poly(acrylic acid)	Lifetime-gated *in vivo* multiplexed imaging
NaGdF_4_: 5%Nd@NaGdF_4_	808	1060	DSPE-PEG-NH2-DNA- FSHβ	Image-guided surgery for metastatic ovarian cancer
NaGdF_4_: 5%Nd@NaGdF_4_	808	−	GSH (Glu–Cys–Gly)	*In vivo* inflammation Imaging
β- NaYF_4_: 7%Nd@ NaYF4	808	1064, 1345	DSPE-mPEG	NIR-II imaging of bone, vascular tissue and thrombi

### Er-Doped Nanoparticles

**Figure 3:**
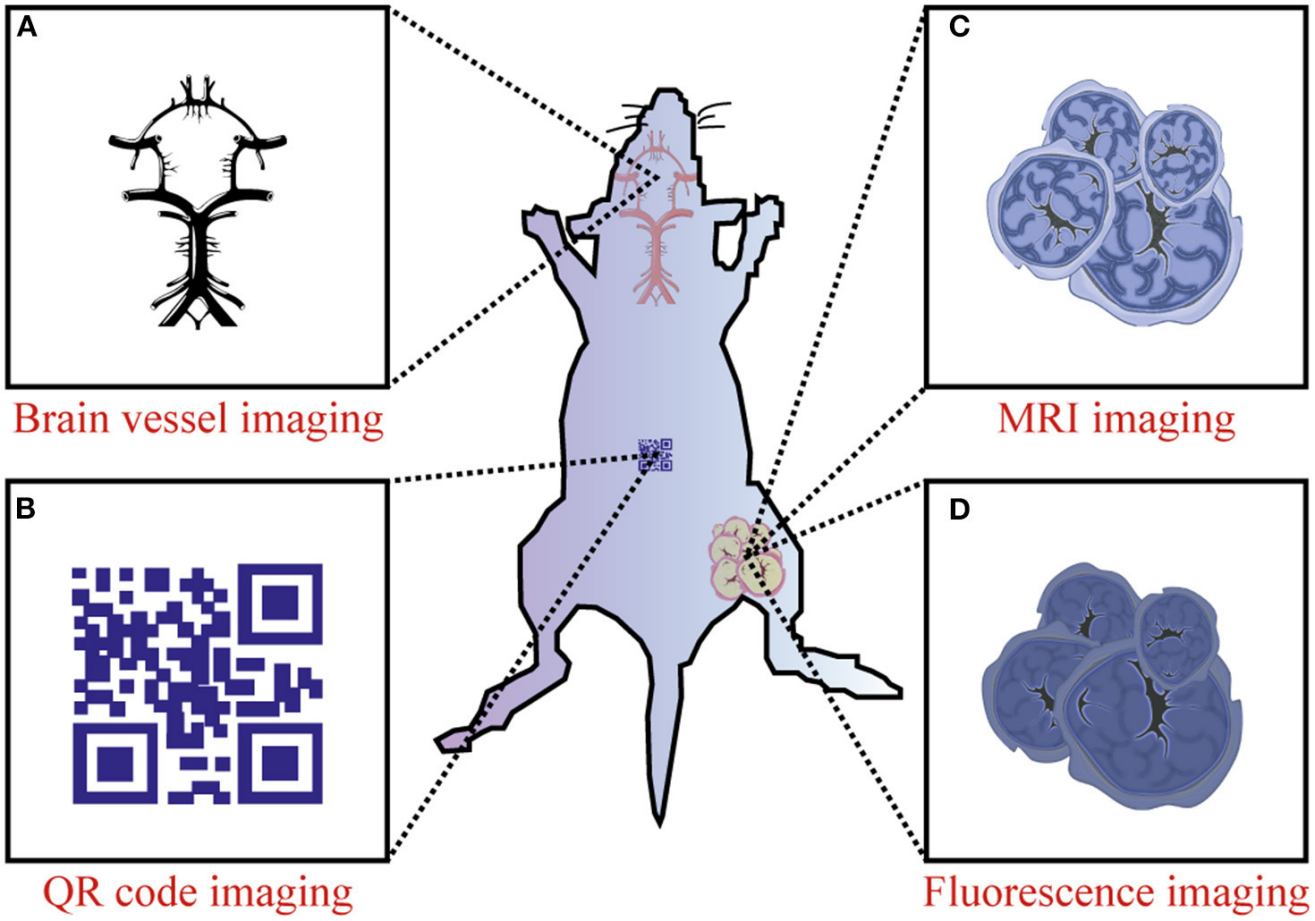
Preclinical application of Er-RENPs in the NIR-II region. **(A)** NIR-II brain vessel imaging, **(B)** NIR-II QR code imaging and **(C,D)** dual mode MRI imaging/ NIR-II fluorescence imaging.

With the rapid development of the RENPs, Er^3+^ doped nanoprobes are mainly synthesized as upconversion nanomaterials and applied in the VIS and NIR-I regions. In 2011, Y_2_O_3_: Yb, Er nanoparticles modified by PEG-b-PVBP and PEG-PO_3_H_2_ showed NIR emission at 1,550 nm in organs of live mice (Kamimura et al., 2011). Then people considered Er^3+^ as a better dopant since it can exhibit strong downconversion luminescence in NIR-IIb region. Nanoprobes employed in the NIR-IIb region are better for bioimaging, owing to their deeper tissue penetration, higher spatial and temporal resolution and lower autofluorescence than those in the NIR-IIa region; but rare-earth based nanoprobes with high spatial and temporal resolution imaged in NIR-IIb region are still very scarce. There is no doubt that the special characteristic of Er^3+^ solves the main problem that has plagued researchers for a long time. Two years later, Naczynski et al. first used NaYF_4_: Yb, Ln (Ln: Er, Ho, Tm or Pr) for *in vivo* imaging of tumors. They demonstrated that Er^3+^ doped nanoprobes were the brightest one. Especially, by encapsulating RENPs with albumin, they provided a good method to improve tumor accumulation (Naczynski et al., 2013). Then, Er^3+^ codoped Yb^3+^ nanoprobes have attracted increasing attention due to their special application potential. Polyacrylic acid (PAA) modified NaYF_4_: Gd/Yb/Er nanoprobes have been synthesized and have opened the opportunities for NIR-IIb *in vivo* imaging, non-invasive brain vessel imaging and tiny tumor detection guided by optical imaging (Xue et al., 2018). In 2016, Dang et al. used the well-established technology, Layer-by-Layer (LbL) to design a NIR-II based theranostic platform by NaYF_4_: Yb, Er-PLA/DXS/PLA/HA nanoprobes, which can accumulate in diseased sites and demonstrate diagnostic capabilities within an ovarian tumor mouse model. This study demonstrated that these nanoprobes can serve as a promising theranostic platform to monitor the progression and treatment of serous ovarian cancer (Dang et al., 2016). Indeed, core-shell is well known for its unique ability to enhance the Er^3+^ emission at NIR-II region. This special structure does not only delay the degradation of dopant but also decreases the quenching effects and strengthens fluorescence. Simple NaYF_4_: Yb/Er@NaYF_4_ nanoprobes have been prepared to realize real-time surveillance of metastatic lesions (Kantamneni et al., 2017). Deng et al. proposed Sc-based probes (KSc_2_F_7_: Yb^3+^/Er^3+^), which are significantly different from the traditional NaYF_4_ host. After modification with PAA, they showed a ~1.70-fold stronger fluorescence than the PAA-NaYF_4_ nanocrystals under 980 nm excitation. On this basis, they performed the first case of through-skull fluorescence imaging of brain vessels with KSc_2_F_7_: Yb^3+^/Er^3+^ probes (Deng et al., 2018). Normally, Yb^3+^ can transfer energy to Er ^4^I_11/2_ level to release non-radiative photons to the ^4^I_13/2_ level, and then radiate to the ^4^I_15/2_ level to produce the 1,550 nm downconversion emission. During this process, upconversion and quenching effects will decrease the intensity of Er^3+^ downconversion emission. As an alternative, Ce^3+^ is developed as a doping element in Er-doped nanoparticles to improve the NIR-II downconversion emission by efficiently accelerating non-radiative relaxation of Er ^4^I_11/2_→^4^I_13/2_. NaYbF_4_: 2%Er, 2%Ce@NaYF_4_ nanoparticles have been made to prove Ce^3+^ can highly suppress the upconversion with the downconversion pathway boosted by about 9-fold. This can lead to fast NIR-II cerebral-vasculatures imaging by modified PMF-PEG (Zhong et al., 2017). The synthesis of NaCeF_4_: Er/Yb@NaCeF_4_ has further verified the efficient energy transfer of Yb^3+^-Er^3+^-Ce^3+^. Surface modification with DSPE-PEG2000-COOH proved to be a useful method to detect uric acid and can be a key approach in a physiological survey and clinical diagnosis (Lei et al., 2018). Interesting research has been done to design and implant QR codes into a mouse by incorporating NaYF_4_: Tm^3+^/Er^3+^@NaYF_4_ into polydimethylsiloxane (PDMS) matrices. The QR code consists of black squares arranged in a square grid on a white background according to certain rules, and the imaging device can read the data from the horizontal and vertical components of the image. It provides a possibility for NIR-II *in vivo* information storage and decoding (Zhang et al., 2019). A core/multishell structure (NaGdF_4_@NaGdF_4_: Yb/Er@NaYF_4_: Yb@NaNdF_4_: Yb) has also been used for breast cancer diagnostics *in vivo* (Fan et al., 2018). Recent progress has focused on the diversification of Er-doped rare-earth nanoparticles. NaYF_4_: Er nanoparticles conjugated with the indocyanine green dye (ICG) have been applied to bioimaging in the NIR-II window because of their high spatial resolution. Due to high absorption cross-section of ICG, excitation efficiency of Er^3+^ is increased by the energy transfer mechanism and has proved the potential of ICG-NaYF_4_: Er nanoconjugates for multimodal theranostics (Wang D. et al., 2018) (Figure 3). Since NIR-II imaging-guided photothermal therapy (PTT) is rarely explored, Liu et al. have successfully developed a core-shell structured NaLuF_4_: Gd/Yb/Er NRs@PDA as a nanoplatform that can simultaneously be used to diagnose and treat tumors. It can not only be used to realize NIR-II imaging but also to enable image-guided PTT (Li X. et al., 2019).

## Synthesis and Modification

**Figure 4:**
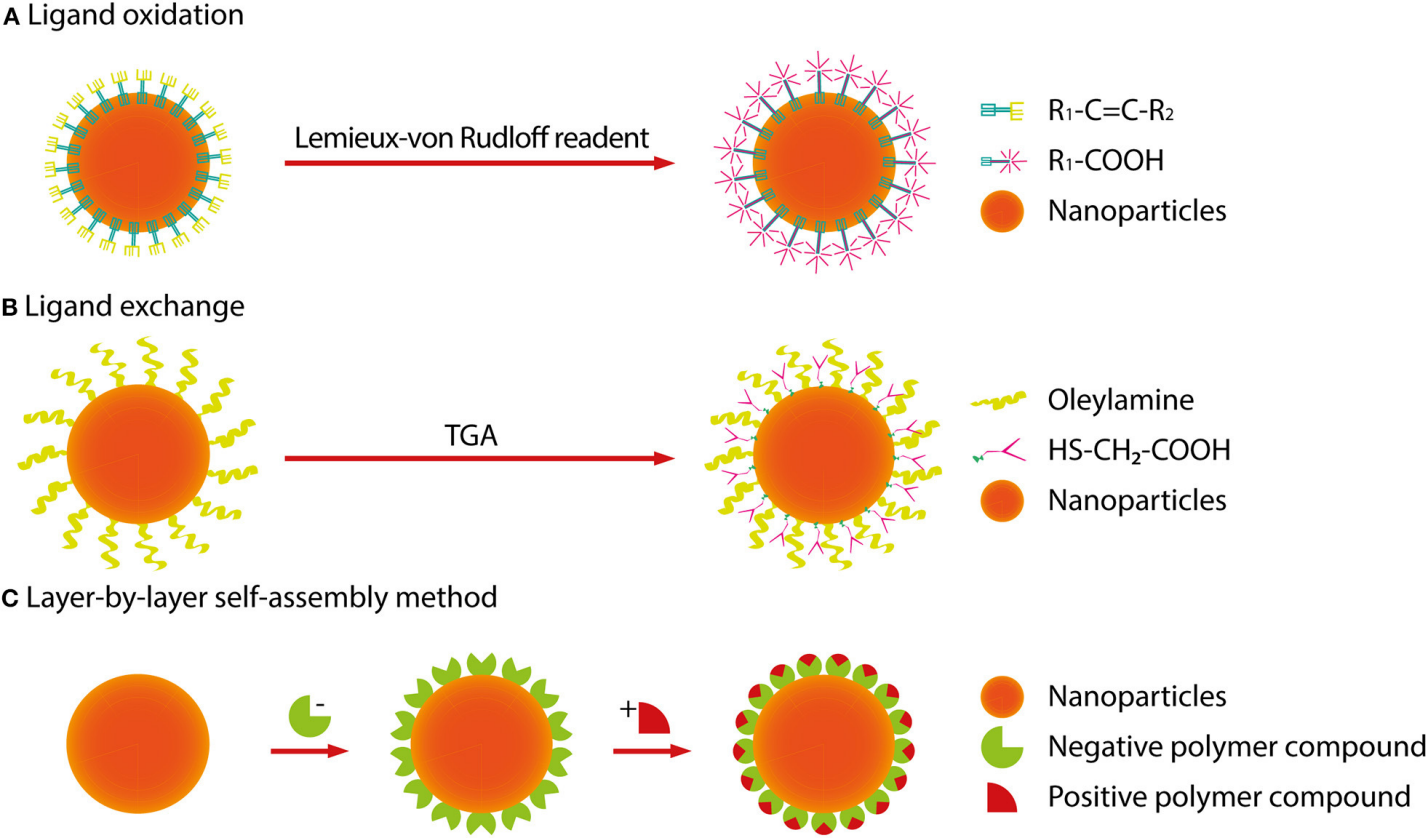
Surface modification methods of RENPs. **(A)** The ligand oxidation method requires a strong oxidizing agent (Lemieux-von Rudloff reagent, etc.) to oxidize the carbon-carbon double bond to a carboxyl group. **(B)** The ligand exchange method is used to replace an organic ligand having weak coordination and hydrophobicity with a strong hydrophilic organic ligand. **(C)** The layer-by-layer self-assembly method relies mainly on the attractive force between oppositely charged molecules, alternately deposits dense monolayers of charged molecules onto oppositely charged surfaces.

At present, RENPs are synthesized by a solvothermal method, which is technically mature. Solvothermal synthesis is a solution chemistry method that crystallizes nanomaterials of different sizes and morphologies directly from solution under a certain temperature and pressure. To synthesize RENPs with uniform size and morphology, good dispensability and high luminous efficiency, the size and morphology have to be controlled by adjusting the ratio of raw materials, temperature and solvents (Mai et al., 2007; Zhang et al., 2007; Tan et al., 2009, 2013; Wang et al., 2010; Yuan et al., 2013). On the other hand, because this process cannot precisely control the distribution of dopants, the local relative enrichment of the dopants usually occurs, resulting in the reduction of luminescent efficiency. In order to avoid the deficiency, one pot successive layer-by-layer (SLBL) strategy is used to synthesize homogeneous doping core (HOC) nanoparticles by growing uniform shells (Li X. et al., 2014, 2019). However, RENPs prepared with oleic acid as reagent are hardly soluble in water and difficult to attach to biomolecules. This limits their application in cell labeling and fluorescent imaging. It is therefore necessary to convert a hydrophobic group into a hydrophilic group by surface modification (for example, -COOH, -NH_2_, or -SH). Alternatively, Dong et al. have reported the oleate ligands attached to the UCNPs surface can be replaced by nitrosonium tetrafluoroborate (NOBF_4_) (Dong et al., 2011). Currently, the main surface modification methods used are ligand oxidation, ligand exchange and layer-by-layer self-assembly (Wang and Liu, 2009; Li and Lin, 2010). The nanoparticle size does not increase after the water-soluble modification using the ligand exchange method, and it is not easy to control the exchange efficiency and effect of water solubility. After using ligand oxidation for modification, due to the shortening of the ligand carbon chain, polar solvent water cannot be effectively suppressed not to quench the fluorescence, and also the fluorescence intensity is much weaker. This method is only suitable for the oxidation of ligands containing carbon-carbon double bonds (C=C). Therefore, it is still a hot Research Topic to select effective water-soluble modification methods to obtain RENPs with small particle size, good water solubility and high fluorescence intensity. The following is a brief summary to the currently used surface modification methods (Figure 4).

### Ligand Oxidation

The ligand oxidation method requires a strong oxidizing agent (Lemieux-von Rudloff reagent, etc.) to oxidize the carbon-carbon double bond to a carboxyl group, which is on the surface-coated oleic acid ligand. This reaction can obtain hydrophilic, carboxylic acid-functional RENPs. Thus, the RENPs not only have good water solubility, but also can be directly coupled with diverse biomolecules.

### Ligand Exchange

The ligand exchange method is used to replace an organic ligand having weak coordination and hydrophobicity with a strong hydrophilic organic ligand on the surface of the material. This process makes the RENPs hydrophilic and water-soluble.

### Layer-by-Layer Self-Assembly Method

The principle of the layer-by-layer self-assembly method (Wang et al., 2002) is to first wrap a layer of polymer compound with a certain charge on the surface of hydrophobic RENPs. When it is added to a solution of an opposite charged polymer compound, another opposite charged polymer compound can be attracted to the first layer. In this way, the layers are adsorbed, and the upper fluorescent group can be converted, which is possible by alternating self-assembled layers into a polymer layer on the surface of the RENPs. The thickness of the polymer layer can be regulated by changing the number of self-assembled layers so that the RENPs can be stably dispersed in water and have good biocompatibility.

## Cancer Theranostics With NIR-II RENPs

Currently, clinically approved indocyanine green (ICG) and methylene blue (MB) have been used as contrast agents to depict tumor margins in preclinical cancer models and human patients (Winer et al., 2010; Vahrmeijer et al., 2013; Wang et al., 2015). However, these rapidly excreted probes possess short tumor retention times and affect the process of cancer treatment. Due to the renal filtration threshold of ~40 kD, most fluorescent probes accumulate largely in the organs of reticuloendothelial system, such as the liver and spleen, leading to long-term safety concerns. Therefore, NIR-II RENPs probes with long tumor retention times, high signal-to-background ratio and deep tissue penetration have aroused great interest in investigating their applications for cancer theranostics. To reduce the retention time of nanoparticles in the reticuloendothelial system, excretable NIR-II nanoparticles, RENPs@Lips, have been developed for medical imaging and surgical navigation. Under the guidance of NIR-II imaging, RENPs@Lips showed excellent performance in intraoperative identification of orthotopic tumor vessels and embolization surgery, and could be used in sentinel lymph node biopsies in tumor-bearing mice (Li D. et al., 2019). CXCR-4-targeted functional nanoprobes (fReANC) have been demonstrated to detect up to 10.5 mm of deep-seeded subtissue microlesions in lung metastatic models of breast cancer, providing a reliable platform for the detection of targeted subtissue cancerous lesions (Zevon et al., 2015). At the same time, Dang et al. compared several available LbL NIR-II probes, found that rare-earth-based down-conversion nanoparticles can define vascular and skeletal structures, and were evaluated as diagnostic probes for high-grade serous ovarian cancer with the highest resolution out of all tested probes (Dang et al., 2016). NIR-II RENPs, with the least interference from scattering and autofluorescence, seemed to represent a promising tool for photothermal therapy (PTT) and photodynamic therapy (PDT). He et al. have designed a unique NaGdF_4_: Nd@NaGdF_4_@NaGdF_4_: Yb, Er@NaGdF_4_: Yb@NaNdF_4_: Yb (LDNPs-5) structure by attaching Au25 clusters and poly(ethylene glycol) (PEG) molecules on nanostructure. Under 808 nm light irradiation, the special LDNPs can efficiently kill tumor cells *in vitro* and *in vivo* due to a synergistic effect arising from the combination of PTT effect generated from Nd^3+^ with PDT (He et al., 2016). In addition, a three-layer core-shell-shell nanocomposite (NaYF_4_: Nd^3+^@NaLuF_4_@PDA_18_) showed an excellent PTT effect in ablation tumors (Dai et al., 2017). Recently, dual-mode SWIR imaging and MRI guided PTT was performed in a nude mouse model by using NaErF_4_@NaGdF_4_ (Er@Gd), which can effectively be used to ablate tumors and provide a new way for cancer theranostics (Ma et al., 2018). NaErF_4_@NaYF_4_@NaNdF_4_@Prussian blue (PB) encapsulated in a phospholipid PEG micelle (PEG-CSS@PB) served as an efficient theranostic agent for NIR-II-image guided PTT. In this study, tumors treated with PTT shrank ~12-fold compared with untreated tumors (Wang et al., 2019c). To achieve accurate tumor localization and a high cancer therapeutic efficacy, Liu et al. developed an ultrasmall pH-responsive photothermal gallic acid-iron complex-modified NaDyF_4_: Nd nanoprobe to enhance cancer theranostic by *in situ* aggregation (Liu et al., 2017). In addition, a theranostic nanoparticle based on RENPs has been developed for gene therapy. Polyethylenimine (PEI) coated β-NaY_0.78_F_4_:Yb_0.20_,Er_0.02_@NaYF_4_ was designed to deliver genetic cargo in an *in vitro* cancer model and detected tumor lesions in a lung metastases model of breast cancer. This strategy will make it possible to develop a nanotheranostic platform based on NIR-II RENPs for gene therapy (Zhao Z. et al., 2018). In summary, NIR-II RENPs have great potential in cancer theranostics.

## Discussion

Overall, RENPs are promising candidates for NIR-II biomedical imaging due to their low toxicity, high photostability, deep tissue penetration, and tunable pharmacokinetic behavior. Despite these successful gains, challenges still remain in the bioimaging applications of NIR-II RENPs. One of them is the limitation of the emission center, which is the fluorescence core of the RENPs. As we know, five rare earth elements (Nd^3+^, Tm^3+^, Pr^3+^, Ho^3+^, Er^3+^) can be the emission centers in NIR-II RENPs, which are excited by 808 nm or 980 nm lasers. However, the RENPs that have been developed are still mainly based on Nd^3+^ and Er^3+^ as the emission centers, which greatly limits the development and application of near-infrared probe types. Although other rare earth elements have also been presented, such efforts should be devoted to design novel NIR-II RENPs probes. For example, Liu et al. used Er^3+^ as a sensitizer and Ho^3+^ as an emitter to make a core-shell structured NaErF_4_: Ho@NaYF_4_ nanoparticle, which emitted at 1,180 nm (Liu et al., 2018). Besides that, the size of the RENPs has always been a concern in bioimaging. Smaller size nanoparticles can effectively enter biological tissues, even cells, but the luminescence intensity of nanomaterials will decrease. Although the commonly used core-shell structure can enhance the luminescence intensity, it will increase the size, making it difficult for the nanoparticles to gain entry into biological tissues and the digestion time will become longer. Designing suitable size nanoparticles is still an essential task to promote the NIR-II bioimaging applications of RENPs. All of these studies in the past decades have pointed out that RENPs will play an important role in drug delivery tracking and multispectral molecular imaging in the near future.

## Author Contributions

ZY, CE, and LC structured the review. ZY wrote the review. CE and LC revised the text. All authors contributed to the article and approved the submitted version.

## Conflict of Interest

The authors declare that the research was conducted in the absence of any commercial or financial relationships that could be construed as a potential conflict of interest.
